# The Life Course Dynamics of Affluence

**DOI:** 10.1371/journal.pone.0116370

**Published:** 2015-01-28

**Authors:** Thomas A. Hirschl, Mark R. Rank

**Affiliations:** 1 Department of Development Sociology, Cornell University, Ithaca, New York, United States of America; 2 Herbert S. Hadley Professor of Social Welfare, George Warren Brown School of Social Work, Washington University, St. Louis, Missouri, United States of America; Leibniz Institute for Prevention Research and Epidemiology (BIPS), GERMANY

## Abstract

Social science research finds that the only group to have experienced real economic gains over the past four decades is the top 20 percent of the income distribution. This finding, along with greater awareness of growing inequality, has renewed interest in mobility research that identifies how individuals and their progeny move into and out of upper versus lower income categories. In this study a new mobility methodology is proposed using life course concepts and life table statistical techniques. Panel data from a prospective national sample of the U.S. population age 25 to 60 are analyzed to estimate the extent of mobility associated with top percentiles in the income distribution. Empirical results suggest high mobility associated with top-level income. For example, 11 percent of the population is found to occupy the top one percentile for one or more years between the ages of 25 and 60. The study findings suggest that many experience short-term and/or intermittent mobility into top-level income, versus a smaller set that persist within top-level income over many consecutive years. Implications of the findings are discussed in terms of inequality buffering, opportunity versus insecurity, and the demographics of income inequality.

## Introduction

Much has been written in recent years about the rising levels of economic inequality in the United States [1]. Research has indicated that the overall gap between the top and bottom of the income distribution has been getting wider [2]. In particular, there has been concern about the fact that the only group to have experienced real economic gains over the past four decades has been the top 20 percent of the income distribution, with such gains heavily concentrated in the top 10^th^, 5^th^, and 1^st^ percentiles [3]. Capturing this concern in the popular media was the Occupy Movement in 2011 and 2012 that focused on the 1 and 99 percent of the income distribution.

Yet the picture drawn of the 1 percent has often been that of a static population, just as the 99 percent is frequently portrayed as unchanging [4]. However, is it the case that the top 1 percent of the income distribution are the same people year in and year out? Or for that matter, what about the top 5, 10, and 20 percent? To what extent do Americans experience such levels of affluence at least some of the time? In this article we explore these questions.

### Background

There are several different ways of thinking about upward income mobility. One approach is what is known as relative mobility. Relative mobility looks at how well children are doing as adults relative to where they were starting with respect to their parents’ economic position within the overall income distribution [5–7]. The vast majority of this research has focused on fathers and sons.

One statistic often used in this body of research has been the intergenerational elasticity statistic. It ranges from 0 to 1, with the higher the number, the stronger the association. Corak [8] has examined dozens of research studies across a number of countries to come up with a comparative analysis of how this statistic varies internationally. The United States is at the high end at .47, indicating that nearly half of the father’s economic position is handed down to his son. On the other hand, Canada, Finland, Norway, and Denmark are the countries with the weakest associations between fathers’ and sons’ incomes, ranging from .19 for Canada, down to .15 for Denmark.

Another measure of relative mobility is what is known as a mobility table. Here the approach is to divide the income distribution into fifths or quintiles. One can then examine which quintile sons fall into with respect to their income, compared to the income quintile that their fathers fell into. By using this approach one can observe to what extent sons are able to rise, fall, or stay roughly the same with respect to their father’s overall economic position.

Jantti et al. [9] have used this approach to show that in the United States, for sons who grew up in households where their fathers fell into the bottom 20 percent of the income distribution, 42.2 percent of such sons remained in the bottom 20 percent of the income distribution as adults. This percentage was much higher than in the other countries examined. In addition, only 7.0 percent of sons growing up in the bottom 20 percent find themselves in the top 20 percent as adults. Conversely, 36 percent of sons who grew up in the top 20 percent remained there as adults, whereas 9.5 percent fell to the bottom 20 percent.

A second broad approach to measuring economic movement is through what researchers refer to as absolute mobility. Here the focus is on answering the question: are children doing better than their parents in terms of sheer income? Consequently, after controlling for inflation, will children earn more and do better financially than their parents did?

Issacs [10] [11] has conducted analyses looking at this question. Using the Panel Study of Income Dynamics (PSID) she compared those who were children in 1968 (and observing their family income from 1967 to 1971) with their family income as adults from 1995 to 2002. She found that 67 percent of adult children were in households earning more than their parents earned. When family size differences were adjusted for, 84 percent of children were earning more than their parents.

Finally, research has begun to look at the very top of the income distribution, and analyzing mobility at this top level [12]. These preliminary studies have shown that there is a substantial amount of movement at the top [13]. For example, Auten et al. [14] found that the “percentage of taxpayers remaining in the top 1 percent for five consecutive years averaged 30 percent” (p.171).

Another example of income fluidity can be found in an analysis by Carroll [15]. Using data from the Internal Revenue Service, he showed that between 1999 and 2007, half of those who earned over $1 million a year did so just once during this period, while only 6 percent reported millionaire status across all nine years. Likewise, data analyzed by the Internal Revenue Service [16] showed similar findings with respect to the top 400 taxpayers between 1992 and 2009. While 73 percent of people who made the list did so once during this period, only 2 percent of them were on the list for 10 or more years. These analyses indicate that there is a sizeable amount of turnover and movement within the top levels of the income distribution.

### The Life Course Approach

An alternative method of examining the extent of economic mobility and income dynamics in the United States is through the life course approach. The concept of the life course has had a long and distinguished history within the social sciences. It has proven to be an extremely helpful tool in thinking about the manner in which individual lives unfold. The term itself refers to “social processes extending over the individual lifespan or over significant portions of it, especially [with regard to] the family cycle, educational and training histories, and employment and occupational careers” (p.3) [17].

With respect to income dynamics, the life course approach has been applied primarily to understanding the extent of poverty within the lifetimes of Americans. The work of Rank and Hirschl has demonstrated that the life course risk of poverty and economic insecurity is quite high when compared to the cross-sectional risk. For example, between the ages of 25 and 60, 54 percent of the population will experience at least one year in poverty or near poverty (150 percent below the federal poverty line). In addition, 79 percent of Americans will experience a year of economic insecurity, which includes either using a social welfare program, encountering poverty or near poverty, or the head of household experiencing unemployment [18].

The earlier reported research on relative and absolute mobility has focused on comparing three- to five-year income averages between parents and their adult children. Yet this represents only a small slice of what happens economically to individuals and families across the prime working years. The life course approach looks across much longer stretches of time to determine how much economic movement occurs in the lives of Americans.

Just as the life course approach can be applied to the bottom end of the income distribution, so too can it be applied to the top end. Consequently, one can look across the prime earning years and examine what percentage of the population will experience affluence at different levels. In the present analysis, we look at individuals between the ages of 25 and 60, and whether they will encounter various levels of affluence.

## Materials and Methods

### Data Set

In order to assess the life course dynamics of affluence over time, we utilize the Panel Study of Income Dynamics (PSID). The PSID began in 1968 as an annual panel survey (biennial after 1997) and is nationally representative of the nonimmigrant U.S. population. The longest running panel data set in the United States, the PSID gathers extensive information regarding household income, making it uniquely suited for this study. The PSID initially interviewed approximately 4,800 U.S. households in 1968, which included detailed information on roughly 18,000 individuals within those households. The PSID has since tracked these individuals, including children and adults who eventually broke off from their original households to form new households (e.g., children leaving home, separations, divorce). Thus, the PSID is designed so that in any given year the sample is representative of the entire nonimmigrant U.S. population (for detailed information regarding the PSID sample and its representativeness, see [19–22]).

Throughout the analysis we employ the individual sampling weights to ensure that the PSID sample accurately reflects the U.S. population. Specifically, we utilize the weights assigned to individuals for each given wave to take advantage of the PSID practice of periodically adjusting the weights to account for nonresponse bias [23].

We utilize both the household and individual levels of information from the initial wave of 1968 through 2011. Consequently, we draw upon 44 years of longitudinal information, which translates into several hundred thousand individual years of information embedded in the analysis. As noted earlier, after the 1997 wave, the PSID began interviewing households every other year. A second change occurring in 1997 was that the PSID sample size was reduced for cost management reasons. The original core sample was reduced from approximately 8,500 in 1996 to 6,168 in 1997 [19]. As noted above, the sample weights are used throughout to ensure that the sample continues to represent the overall population and that the reduction in sample size does not bias our estimates [24].

Total family income is the measuring stick used to determine levels of affluence achieved. This is defined as taxable income of head of household and spouse, taxable income of other family members of the household, and transfer income of head, spouse, and others. The PSID questionnaire includes a lengthy set of income questions designed to recover multiple forms of taxable income sources.

PSID total family income estimates were found to be comparable to estimates from the March Current Population Survey over the years 1968 to 2007, for each quintile, as well as for the 95th percentile [25]. When comparing top-level income percentiles based on PSID total family versus income percentiles based on reports to the Internal Revenue Service (IRS) [26], the PSID percentiles are generally higher, perhaps because the PSID’s family definition is demographically more complete relative to IRS “taxable units.” For the 90th percentile, the PSID thresholds are higher than the IRS thresholds in every year between 1967 and 2010 [27]. At the 99th percentile over the same time period, the PSID thresholds are higher than the IRS thresholds in all but three years [27]. Thus we find no evidence that the PSID, in comparison to other reports, is biased toward middle level income, or otherwise fails to fully enumerate top-level income at the 99th percentile and below.

### Life Table Technique

In describing the life course patterns of affluence dynamics over time, we rely upon the life table as our major analytical technique. Life tables are a concise method for describing how the odds of experiencing a specific event change as individuals age over time. The life table is most closely associated with biological and demographic studies of mortality, but can be easily applied to estimate the occurrence of other events as well [28] [29].

In this analysis, our time intervals comprise each year (or two) that an individual ages. During that year, one can calculate the probability of an event occurring (in this case, affluence) for those who have yet to experience the event. Once the event has occurred, the individual is no longer at risk and therefore exits the life table. Based upon these age specific probabilities, the cumulative probabilities of an event occurring across the life course can be calculated. These cumulative probabilities form the core of our analysis.

Individuals may contribute anywhere from 1 to 36 person-years within the life table. For example, a woman within the PSID study who turned 25 in 1975 and then in 1979 experienced a year of affluence would have contributed five person years within our analysis. In this case, she would be included in the estimates for ages 25, 26, 27, 28, and 29. Period effects are smoothed out both within and across the age intervals.

Life tables are calculated for those hitting the top 20^th^ percentile in terms of family income, as well as the top 10^th^ percentile, 5^th^ percentile, and 1^st^ percentile. These percentiles were calculated for each year of the PSID for individuals aged 25 to 60. To measure chronicity for each of these levels, life tables are calculated for individuals that experience one or more years, two or more years, three or more years, four or more years, five or more years, and ten or more years. Finally, calculations are made for consecutive versus total years to identify spell length within each of these levels.

The life table analysis utilizes 44 years of prospective PSID data to compute 36 years of life course probabilities. Sample members enter the analysis at age 25, and are tracked until age 60. Because sample members in PSID waves 1968 through 1975 can reach age 60 by 2011, individuals within eight waves can hypothetically age through the final year of life table analysis. This temporal window provides ample data for a fully prospective analysis in the sense that a large set of individuals are under annual (bi-annual after wave 1997) observation beginning at age 25 through age 60.

## Results

Table 1 contains the cumulative percentage of adults experiencing various levels of affluence between the ages of 25 and 60. We can see that by age 60, 69.8 percent of the population will have experienced at least one year within the top 20^th^ percentile, 53.1 percent will have experienced at least one year within the top 10^th^ percentile, 36.4 percent will have encountered one year within the top 5^th^ percentile, and 11.1 percent will have experienced one year within the top 1^st^ percentile.

**Table 1:** Cumulative Percentage of American Adults Experiencing Various Levels of Household Affluence by Age (Standard Errors in Parentheses).

**Age**	**20 percent**	**Top of Income Distribution**		
		**10 percent**	**5 percent**	**1 percent**
25	11.0 (2.1)	5.5 (1.5)	3.1 (1.2)	0.5 (0.5)
30	28.6 (3.2)	14.9 (2.5)	7.9 (1.9)	1.2 (0.9)
35	43.1 (3.8)	24.3 (3.3)	13.5 (2.6)	3.0 (1.3)
40	53.3 (4.3)	32.8 (4.0)	19.5 (3.5)	5.0 (1.9)
45	61.3 (4.7)	39.8 (4.6)	25.3 (4.1)	6.7 (2.4)
50	66.2 (5.0)	46.5 (5.3)	30.2 (4.8)	8.3 (2.9)
55	69.1 (5.4)	50.8 (5.9)	34.3 (5.6)	10.0 (3.5)
60	69.8 (5.6)	53.1 (6.5)	36.4 (6.2)	11.1 (4.1)
N	146,463	180,889	199,926	221,727

These results indicate that there is a substantial amount of income mobility at these percentile levels. For example, approximately 70 percent of the U.S. population will find themselves for at least one year in the top 20^th^ income percentile. Rather than static groups that experience continual high levels of income attainment, there would appear to be more fluid movement into and out of these income levels.

Table 2 displays the overall number of years of affluence experienced between the ages of 25 to 60. The top panel contains the total number of years, whereas the bottom panel shows the consecutive number of years. In looking at the top panel, we can see that the percentages of the population experiencing greater number of total years declines with the number of years. For example, while 11.1 percent of the population will experience at least one year of income in the top 1^st^ percentile, only 2.2 percent will do so in 5 or more years spread out across the 25 to 60 age interval, and 1.1 percent will do so in 10 or more years.

**Table 2:** Cumulative Percentage of American Adults Experiencing Various Years of Household Affluence (Standard Errors in Parentheses).*

**Years of Affluence**	**Top of Income Distribution**			
	**Years of Affluence**	**20 percent**	**10 percent**	**5 percent**	**1 percent**
**Total Years**				
1 or more	69.8 (5.6)	53.1 (6.5)	36.4 (6.2)	11.1 (4.1)
2 or more	61.8 (6.5)	40.1 (6.4)	26.2 (5.9)	5.8 (3.1)
3 or more	54.5 (6.7)	34.5 (6.3)	20.4 (5.5)	4.2 (2.7)
4 or more	49.9 (6.8)	29.7 (6.2)	15.8 (4.9)	2.9 (2.2)
5 or more	46.0 (7.0)	26.0 (6.2)	12.1 (4.3)	2.2 (2.0)
10 or more	31.4 (7.0)	14.2 (5.3)	6.6 (3.7)	1.1 (1.4)
**Consecutive Years**				
1 or more	69.8 (5.6)	53.1 (6.5)	36.4 (6.2)	11.1 (4.1)
2 or more	55.8 (6.4)	35.4 (6.1)	22.2 (5.5)	4.5 (2.7)
3 or more	47.4 (6.6)	26.7 (5.5)	15.4 (4.9)	3.0 (2.2)
4 or more	41.7 (6.7)	21.7 (5.3)	11.3 (4.5)	2.4 (2.0)
5 or more	35.3 (6.7)	18.2 (5.2)	8.1 (3.6)	1.6 (1.7)
10 or more	20.6 (6.2)	7.8 (3.9)	3.7 (2.8)	0.6 (1.0)

*The N for each of the life table estimates varies between 146,463 and 227,300 person years.

The bottom panel of Table 2 displays the percentage of the population experiencing multiple consecutive years at particular income levels. These percentages are lower than the total number of years. Thus, 20.6 percent of the population will experience 10 consecutive years within the top 20^th^ percentile, 7.8 percent will do so in the top 10^th^ percentile, 3.7 percent will do so in the top 5^th^ percentile, and 0.6 percent will do so in the top 1^st^ percentile.

The results in Table 2 confirm the proposition that attaining top-level income is relatively prevalent in the United States. Consecutive years are less common than total years, in particular for the top 1 percent and top 5 percent levels. Attaining 10 consecutive years at the top 1^st^ percentile is rare (0.6 percent), and reflects the idea that only a few persist at this elite level. When compared to the 11.1 percent that ever achieve top 1 percent income, the findings indicate high year-to-year turnover within this category.

In Table 3 we look at the occurrence of affluence over separate 10-year age intervals across the life course. We therefore start the life table analyses at the beginning of each 10-year age interval. In general, research has shown that affluence is more likely to occur during the later periods of the prime working years. We can see from Table 3 that those most likely to hit a year of affluence are in the prime earning years of 45 to 54. Consequently, 55.8 percent of those 45 to 54 will experience at least one year of household income in the top 20^th^ percentile, 37.9 percent will be in the top 10^th^ percentile, 23.5 percent in the top 5^th^ percentile, and 6.3 percent in the top 1^st^ percentile.

**Table 3:** Cumulative Percentage of American Adults Experiencing Various Levels of Household Affluence Across Age Categories (Standard Errors in Parentheses).

**Age Category**	**20 percent**	**Top of Income Distribution**		
		**10 percent**	**5 percent**	**1 percent**
25–34	40.6 (3.7)	22.4 (3.2)	12.6 (2.5)	2.7 (1.3)
N	101,342	114,959	120,582	125,332
35–44	51.0 (3.9)	31.2 (3.6)	19.2 (3.1)	4.7 (1.7)
N	74,381	86,598	93,148	98,922
45–54	55.8 (4.0)	37.9 (3.9)	23.5 (3.4)	6.3 (2.0)
N	52,172	62,476	69,106	75,650
55–64	46.7 (4.4)	30.7 (4.0)	19.4 (3.5)	6.0 (2.2)
N	38,095	43,371	47,002	50,859

In Table 4 we present a multivariate analysis predicting the occurrence of various levels of affluence. The logistic regression odds ratios for each independent variable are found in the table. Age is entered as a continuous variable, whereas the other variables are dichotomous. They include race (white/nonwhite), gender (male/female), marital status (married/not married), education (greater than 12 years/12 years or less) and work disability status (no work disability/work disability).

**Table 4:** Logistic Regression Model Odds Ratios, Coefficients, and Standard Errors Predicting the Occurrence of Various Levels of Affluence Between the Ages of 25 and 60.

**Variables**	**20 percent**	**Top of Income Distribution**		
		**10 percent**	**5 percent**	**1 percent**
Age	1.05^a^ ***	1.05***	1.04***	1.05***
	.044^b^	.045	.043	.044
	.001^c^	.001	.001	.003
White	2.15***	2.38***	3.02***	6.74***
	.765	.866	1.11	1.91
	.015	.023	.036	.122
Male	1.04**	1.06***	1.05*	.934
	.040	.054	.046	−.068
	.013	.017	.023	.052
Married	6.11***	5.49***	4.82***	4.12***
	1.81	1.70	1.57	1.42
	.021	.031	.045	.108
GT 12	2.87***	3.28***	3.72***	4.37***
	1.05	1.19	1.31	1.48
	.013	.018	.027	.068
No Disability	2.56***	2.22***	2.07***	1.94***
	.938	.797	.725	.663
	.034	.047	.067	.158
N	177,927	177,927	177,927	177,927

^a^Odds ratio.

^b^Unstandardized regression coefficient.

^c^Standard error of regression coefficient.

*significant at the .05 level

**significant at the .01 level

***significant at the .001 level

We can see that with the exception of gender, all independent variables are significantly associated with attaining various levels of affluence. Those who are older, white, married, with greater than 12 years of education, and who do not have a work disability, are significantly more likely to encounter a year of affluence.

## Discussion

Social awareness of the growing distance between top-level earners versus the rest of the income distribution helped to spark the Occupy movement and focus media attention on economic inequality. Much of the associated rhetoric presumes that the same individuals persist in top-level percentiles, in particular the 1 percent. This presumption is erroneous to the extent that year-to-year mobility functions to turnover incumbents. To the extent there is turnover, then this functions to buffer inequality, e.g. take the hypothetical case of 100 percent annual turnover within the composition of the top 10 percent, creating the condition of no inequality at this percentile level when measured across a decade. This study explores this empirical possibility, and other possibilities, by analyzing mobility associated with top-level income in the United States.

The paper has several strengths and weaknesses. First, the longitudinal study design avoids the limitation of truncated income observation, an important advantage to the extent that there is substantial annual variation in family income. In the present study family income is observed for each year of the study, and the study findings reflect observed income over 36 years of the life course between ages 25 and 60. Second, life table techniques are used to compute lifetime income attainment, and these techniques yield robust life course estimates.

Weaknesses include a lack of sufficient sample size representing the U.S. immigrant population, and failure to describe and test a causal explanation related to the study objectives. Our aim, however, is not to identify causality related to top-level income attainment, but rather to describe its pattern with respect to time.

The study is summarized in terms of four findings. First, that there is substantial fluidity in top-level income over ages 25 to 60. The study findings suggest that lifetime rates of top-level income are multiples of the percentile category, e.g. the lifetime number of individuals attaining top 1^st^ percentile income is 11 times the number who attain it within any given year; the lifetime number of individuals attaining top 5^th^ percentile income is 7 times the number attaining it within any given year (see Table 1). Thus a static image of top-level income tenure is at odds with the empirics of how people live out their life course.

Second, the study findings indicate that top-level income categories are heterogeneous with respect to time, comprised of a relatively small set of persistent members, and a larger set of short-term members. For example, although over half of the U.S. population experienced one or more years of top 10^th^ percentile income, only about half of this set attained top 10^th^ percentile income for three consecutive years, and less than 7 percent persisted at this level for 10 consecutive years. Thus the lifetime top 10^th^ percentile is mostly transitory, moving in and out of this percentile over the life course.

Third, there are two contentious social implications related to the finding that top-level income is fluid across time. One is that there is widespread opportunity for top-level income. The opportunity to attain top-level income is widely accessed, and many reap the benefits of opportunity. It is also the case that attaining top-level income in one year does not necessarily predict it for the following year. Indeed, most who attain top-level income do so for a limited number of years, and to the extent that they have expectations of persistence, have some probability of experiencing insecurity relative to their expectations. Income fluidity is a double-edged sword, creating opportunity for many, along with insecurity that this opportunity may end sooner than hoped for.

The demographic pattern of who gets top-level income is familiar and expected for researchers of U.S. social and economic inequality. Education, marriage, and race are among the strongest predictors of top-level income, and in particular the race effect suggests persisting patterns of social inequality related to past and present discrimination and exclusion. Thus it would be misguided to presume that top-level income attainment is solely a function of hard work, diligence, and equality of opportunity. A more nuanced interpretation includes the proposition that access to top–level income is influenced by historic patterns of race and class inequality.

Finally, we interpret the widespread attainment of top-level income as materially consistent with the way the majority of Americans tend to characterize their society. In a recently published study [18], we report evidence that most Americans hold fast to the belief that hard work will be rewarded economically, and the present study finds evidence that many Americans do, in fact, attain top-level income. This evidence is counter-intuitive vis-à-vis popular interpretations regarding the 1 percent versus the 99 percent, and we believe that our findings serve to qualify these interpretations. When interpreting social and economic relationships and trends, it is important to consider not simply one, or even many, cross-sections in time, but also the extent of social and economic mobility across the life course. Individuals experience their lives not as a disconnected set of years, but rather as a continuous lifetime of experience.
